# Hybrid Adeno-Associated Viral Vectors Utilizing Transposase-Mediated Somatic Integration for Stable Transgene Expression in Human Cells

**DOI:** 10.1371/journal.pone.0076771

**Published:** 2013-10-08

**Authors:** Wenli Zhang, Manish Solanki, Nadine Müther, Melanie Ebel, Jichang Wang, Chuanbo Sun, Zsuzsanna Izsvak, Anja Ehrhardt

**Affiliations:** 1 Max von Pettenkofer-Institute, Department of Virology, Ludwig-Maximilians-University Munich, Munich, Germany; 2 Institute of Virology and Microbiology, Center for Biomedical Education and Research, Department of Human Medicine, Faculty of Health, University Witten/Herdecke, Witten, Germany; 3 Max Delbrück Center for Molecular Medicine, Berlin, Germany; National Institute of Dental and Craniofacial Research, United States of America

## Abstract

Recombinant adeno-associated viral (AAV) vectors have been shown to be one of the most promising vectors for therapeutic gene delivery because they can induce efficient and long-term transduction in non-dividing cells with negligible side-effects. However, as AAV vectors mostly remain episomal, vector genomes and transgene expression are lost in dividing cells. Therefore, to stably transduce cells, we developed a novel AAV/transposase hybrid-vector. To facilitate SB-mediated transposition from the rAAV genome, we established a system in which one AAV vector contains the transposon with the gene of interest and the second vector delivers the hyperactive Sleeping Beauty (SB) transposase SB100X. Human cells were infected with the AAV-transposon vector and the transposase was provided in trans either by transient and stable plasmid transfection or by AAV vector transduction. We found that groups which received the hyperactive transposase SB100X showed significantly increased colony forming numbers indicating enhanced integration efficiencies. Furthermore, we found that transgene copy numbers in transduced cells were dose-dependent and that predominantly SB transposase-mediated transposition contributed to stabilization of the transgene. Based on a plasmid rescue strategy and a linear-amplification mediated PCR (LAM-PCR) protocol we analysed the SB100X-mediated integration profile after transposition from the AAV vector. A total of 1840 integration events were identified which revealed a close to random integration profile. In summary, we show for the first time that AAV vectors can serve as template for SB transposase mediated somatic integration. We developed the first prototype of this hybrid-vector system which with further improvements may be explored for treatment of diseases which originate from rapidly dividing cells.

## Introduction

Gene therapy is a rapidly developing field relying on introduction of nucleic acids into mammalian cells to regulate, repair, replace, add or delete a genetic sequence. Monogenetic diseases like hemophilia B, Duchenne muscular dystrophy and cystic fibrosis are the three most frequent indications for clinical trials in gene therapy [1]. For life-long correction of genetic diseases, therapeutic DNA needs to be efficiently delivered to the respective target tissue and cells and transgene expression needs to be maintained at a therapeutic level.

Adeno-associated virus (AAV) belongs to the family of parvoviridae and contains a single-stranded DNA genome of about 4.7 kilobases (kb) in length. Its genome is flanked by inverted terminal repeats (ITR) and encodes the two major open reading frames (ORFs) *rep* and *cap* [2]. Known encoded proteins of *rep* include Rep78, Rep68, Rep52 and Rep40 and *cap* encoded proteins include VP1, VP2 and VP3, and the assembly-activating protein AAP. Recombinant AAV vectors lack both ORFs and combine several advantages, including efficient infectivity, stable transgene expression in quiescent cells and nonpathogenicity [3]. AAV vectors have been extensively investigated in preclinical and clinical settings [4] and they were involved in several clinical trials to treat metabolic abnormalities, hemophilia disease, Parkinson’s disease, muscular dystrophy and cystic fibrosis [2,4,5]. Towards this end several AAV serotypes were explored showing different tropisms in vivo [6] which significantly extended applications of AAV vectors for clinical and other applications.

After in vivo administration, AAV vectors can result in efficient and long-term transduction of non-dividing cells. However, as AAV vectors mostly remain episomal, vector genomes and transgene expression are lost over time in dividing cells [7]. Therefore, to stably transduce tissues and cells undergoing cell division, genetic elements for maintenance of therapeutic DNA need to be combined with the AAV technology for efficient long-term transgene expression. In the present study, we developed a novel AAV/transposase hybrid-vector for somatic integration of the genetic payload from the AAV vector genomes into the host chromosomes utilizing the Sleeping Beauty (SB) transposase integration machinery. The SB transposase system represents a powerful tool for somatic integration and it was demonstrated that it has fundamental implementations for experimental and therapeutic gene transfer approaches [8,9]. The transposable element SB has been generated from inactive copies of an ancestral Tc1/mariner-like transposon in fish [8]. In the presence of transposase supplied in trans, any gene of interest flanked by inverted repeats (IRs) represents a substrate for transposition resulting in somatic integration into a TA-dinucleotide [8,10]. Very recently hyperactive SB transposase versions HSB5 [11] and SB100X [12] were generated by mutagenesis screens which resulted in 10- and 100-fold increased integration efficiencies, respectively. Previous data suggest that the target sites of integration after SB mediated recombination show a close to random genomic distribution profile. Based on studies utilizing different delivery vehicles for the SB transposase system, it was estimated that 39-53% of transposition events are located in genes [13-15].

Herein, we aimed at establishing AAV vectors for stabilized transgene expression in mammalian cells. We show for the first time that AAV vectors can serve as template for SB transposase mediated somatic integration with a close to random integration profile.

## Results

### AAV vectors serve as direct substrates for transposition

**Figure 1:**
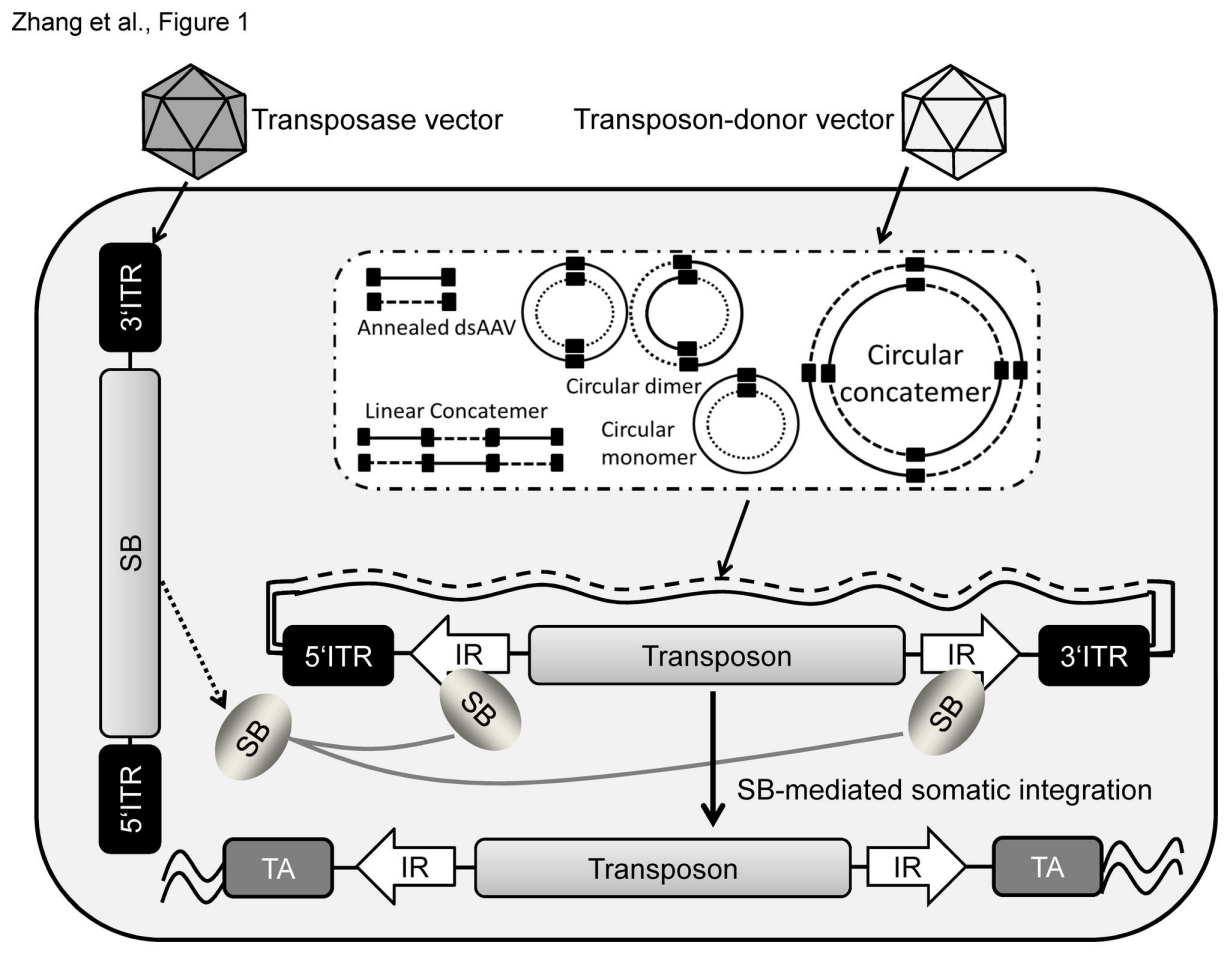
Principle of the hybrid-vector system based on Sleeping Beauty (SB) transposase-mediated transposition from AAV vector genomes. For somatic integration cells were simultaneously infected with the transposon-donor vector and the SB transposase encoding virus. After entering the cell, single-stranded AAV vector genomes form double-stranded DNA and generate several different molecular forms including circular monomers, dimers, and concatemers as well as linear monomers and concatemers [16]. Next, the transposon flanked by inverted repeats (IR, white horizontal arrow) and the AAV-derived inverted terminal repeats (ITR, black squares) needs to be mobilized from the various AAV vector genome forms. The transposon is then integrated into the host genome by a cut-and-paste mechanism mediated by the SB transposase protein which is delivered in trans and encoded by the second vector. Subsequently the transposon is integrated into chromosomal DNA (waved line) into the genomic target site (TA dinucleotide, grey square).

After cellular transduction, AAV vector genomes form various DNA forms such as episomal circular and linear monomers and concatemers [16]. For achieving stabilized transgene expression the goal of this study was to mobilize a transposon from episomal AAV vector genomes for SB transposase-mediated stable integration of a transgene expression cassette into the mammalian host genome (Figure **1**).

**Figure 2:**
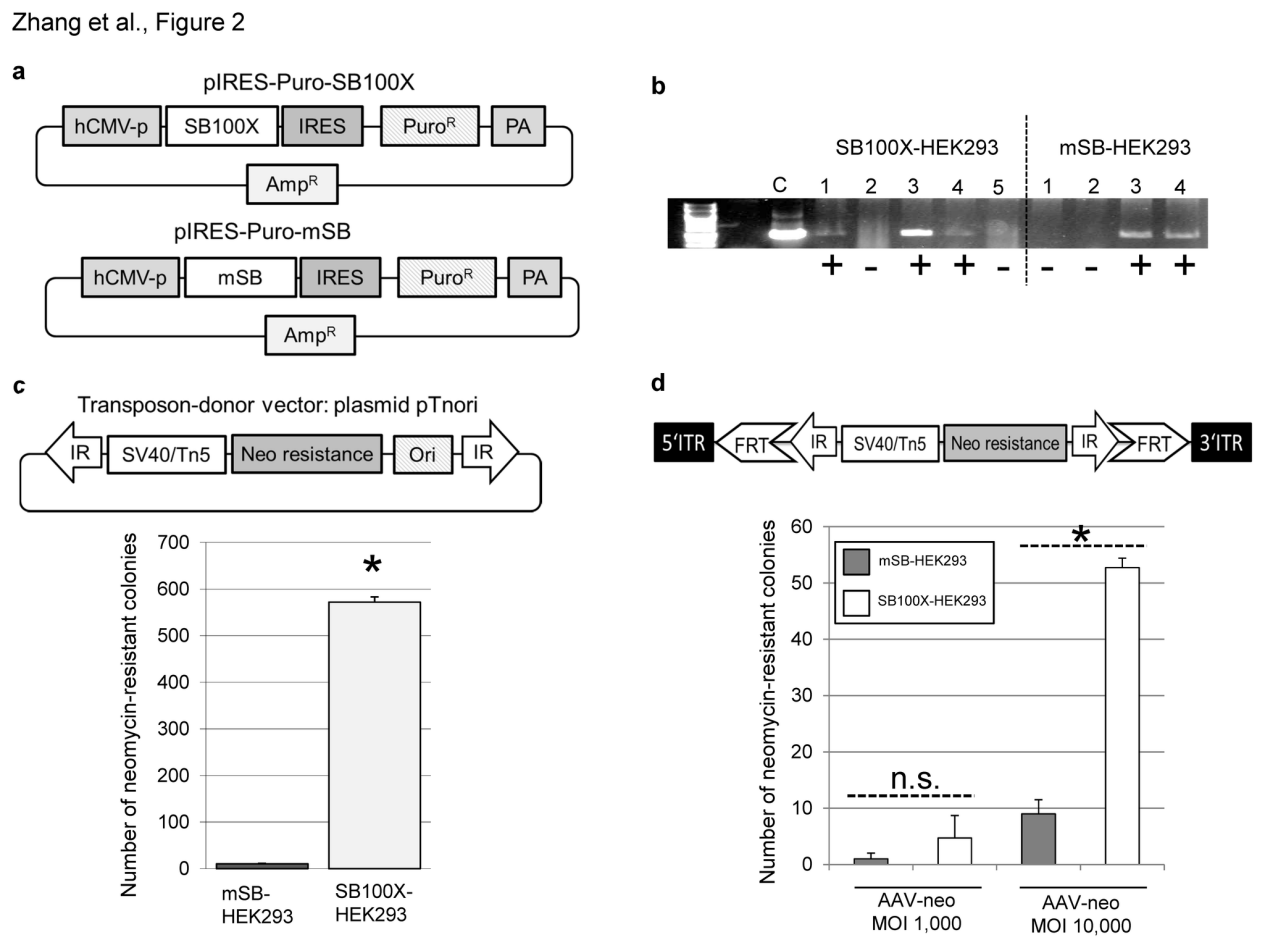
Transposition efficiencies in HEK293 cells stably expressing hyperactive Sleeping Beauty transposase SB100X. (**a**) Plasmids pIRES-Puro-SB100X and pIRES-Puro-mSB were generated containing a bicistronic IRES construct expressing SB100X or mSB and the puromycin resistance gene under the control of the cytomegalovirus promoter (CMV). (**b**) Cells were stably transfected with plasmids pIRES-Puro-SB100X or pIRES-Puro-mSB and single cell clones were amplified under puromycin selection pressure. Total RNA was isolated from single cell clones and RT-PCR was performed to show expression of SB100X and mSB using primers SB100X-rev and SB100X-forw (Table S1). Clone 3 from the selected SB100X and mSB cell clones was chosen for further experiments. (+): Cell clones which stably express SB100X or mSB. (-): Cell clones which were negative for transposase expression. (**c**) The previously published plasmid pTnori [10] containing a neomycin encoding transposon was transfected into stably expressing SB100X and mSB (SB100X-HEK293 and mSB-HEK293) cells and kept under selection pressure using neomycin to select for transposition events. Obtained cell colonies were stained with methylene blue and counted. Error bars indicate standard deviation (n=3). *Significant difference between the SB100X and the mSB control groups (p-value < 0.05). (**d**) SB100X-HEK293 and mSB-HEK293 cells were infected with the recombinant vector AAV-neo at different MOIs (MOIs 1,000 and 10,000). AAV-neo represents the transposon-donor vector from which the transposon is mobilized. The transposon is flanked by Sleeping Beauty transposase derived inverted repeats (IR) and it expresses the neomycin resistance gene under control of the simian virus promoter (SV40) for eukaryotic expression and the Tn5 promoter for expression in bacteria. Additionally the transposon is flanked by the Flpe recognition sites FRT. Error bars indicate standard deviation (n=3). *Significant difference between the SB100X and mSB control groups (p-value < 0.05), “n.s.”: not significant, no significant difference between the SB100X and the mSB control groups (p-value > 0.05).

To assure that all cells express hyperactive SB transposase SB100X, we first established cell lines stably expressing either SB100X or inactive SB transposase (mSB). These cell lines were generated using stable transfection of plasmids pIRES-Puro-SB100X and pIRES-Puro-mSB (Figure **2a**). As shown in Figure **2b**, these cells were tested for transposase expression on RNA level by reverse transcription and subsequent PCR analysis. After transfection of the transposon donor plasmid pTnori and selection of stably transduced cells, SB100X expressing cells showed a significantly increased number of colony forming units proving functionality of SB100X in these cells (Figure **2c**). To perform respective experiments with AAV vectors, we produced the AAV vector AAV-neo containing a transposon with a neomycin resistance gene for expression in mammalian cells and bacteria (Figure **2d**). This vector also contained an origin of replication allowing integration site analysis based on a plasmid rescue protocol. Stably expressing SB100X and mSB cells were then infected with AAV-neo at MOI 1,000 and MOI 10,000. After performing a colony forming assay including two weeks of selection pressure we observed 6-fold increased integration efficiencies when using the SB100X expressing cell line infected with AAV-neo MOI10,000 (Figure **2d**).

**Figure 3:**
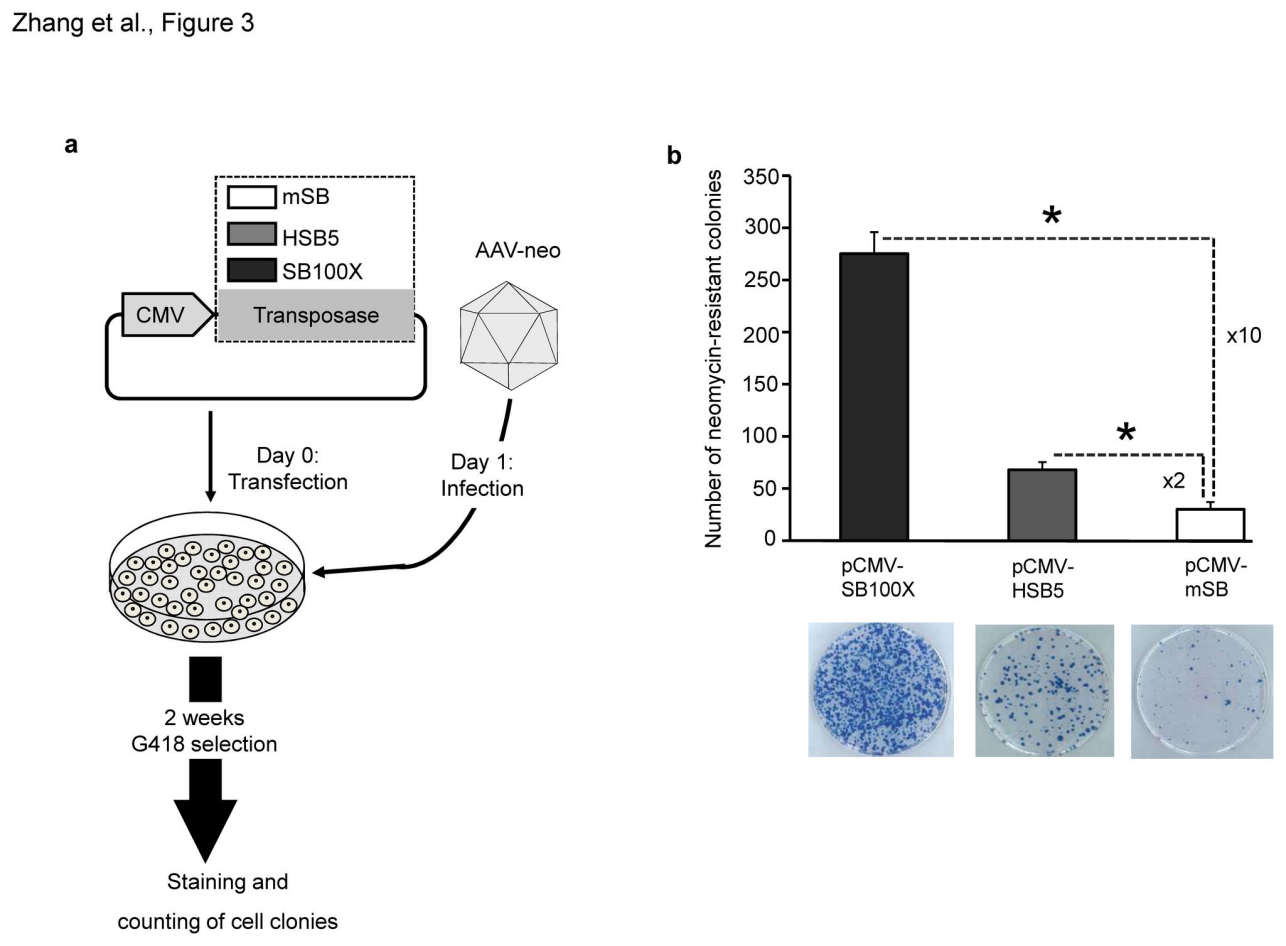
Transposition efficiency after infection with the transposon-donor vector AAV-neo co-transfected with transposase encoding plasmids. (**a**) Colony forming assay to determine integration efficiencies from the AAV-neo vector co-transfected with transposase encoding plasmids. HeLa-cells were first transfected with 1µg of the respective transposase encoding plasmid (pCMV-mSB, pCMV-HSB5, or pCMV-SB100X) and one day post-transfection cells were infected with AAV-neo at MOI 10,000. Two days post-infection cells were diluted and kept under selection pressure for 14 days. Obtained cell colonies were either collected as cell pools for integration site analysis or stained with methylene blue to determine transposition rates. Transposase encoding plasmids contain expression cassettes for the hyperactive transposases HSB5 and SB100X and the inactive SB transposase version mSB expressed under the control of the cytomegalovirus promoter (CMV). (**b**) Result of the colony forming assay. The Y-axis shows the number of neomycin resistant colonies obtained from 4 x 10^5^ cells in different experimental settings. The lower panel shows examples of original tissue culture plates from the different groups after methylene blue staining. Error bars indicate standard deviation (n=3). *Significant difference between the hyperactive transposase groups (SB100X and HSB5) and the mSB control group (p-value < 0.05).

To provide SB transposase in a transient manner, HeLa-cells were transfected with plasmids pCMV-HSB5, pCMV-SB100X or pCMV-mSB encoding the previously described hyperactive transposases HSB5 [11], SB100X and the mutated and inactive transposase mSB. Twenty-four hours post-transfection all groups were infected with the transposon-donor vector AAV-neo (Figure **3a**). After performing a colony forming assay by keeping cells under selection pressure for two weeks, we found that the groups which received the hyperactive SB transposases SB100X and HSB5 resulted in 10-fold and 2-fold increased number of colony forming units compared to the control group which received the plasmid pCMV-mSB (Figure **3b**). Due to the low transposition efficiencies of HSB5 in the context of an AAV vector, we focused our further studies on the hyperactive SB transposase version SB100X.

Our former studies revealed that predominantly circular DNA molecules can serve as substrates for transposition [9]. Therefore, we also addressed the question whether circularization of the transposon-donor as circular monomer from the various episomal molecular forms of the AAV vector may increase transposition (Figure **S1**). To form circular monomers of the transposon we applied the Flpe/FRT recombination system for excision of the transposon from the AAV vector (Figure **S1**). Towards this end we generated AAV vector plasmids which after co-transfection can release circular transposons from the linear AAV genome. In detail, the transposon-donor plasmid pAAV-neo contains FRT sites flanking the neomycin encoding transposon (Figure **2d**). The transposase encoding vectors either encode Flpe recombinase and SB transposase (pAAV-SB100X-Flpe) or solely SB transposase (pAAV-SB100X) (Figure **S2a**). We co-transfected the respective transposon-donor plasmid and one of the transposase encoding plasmids either with or without a Flpe encoding sequence. After performing colony forming assays we found that transposition efficiencies actually decreased in the presence of Flpe recombinase (Figure **S2b**). To perform respective experiments with AAV vectors, we co-transduced SB100X stably expressing cells (Figure **2**) with the transposon donor vector AAV-neo and the previously described Flp encoding high-capacity adenoviral vector Ad5-mSB-Flpe [9] to provide Flpe recombinase in trans. After co-infection, Flp recombinase first mobilizes the transposon from the AAV genome which can then be integrated into the host genome via SB-mediated transposition (Figure **S1**). As shown in Figure **S3** transposition efficiency is independent of Flp expression supplied by Ad5-mSB-Flpe. Based on these studies we decided to exclude Flpe-mediated excision of the transposon substrate from the AAV vector DNA molecule.

### Transposition efficiencies after co-delivery of the transposon-donor AAV vector and the transposase encoding AAV vector

**Figure 4:**
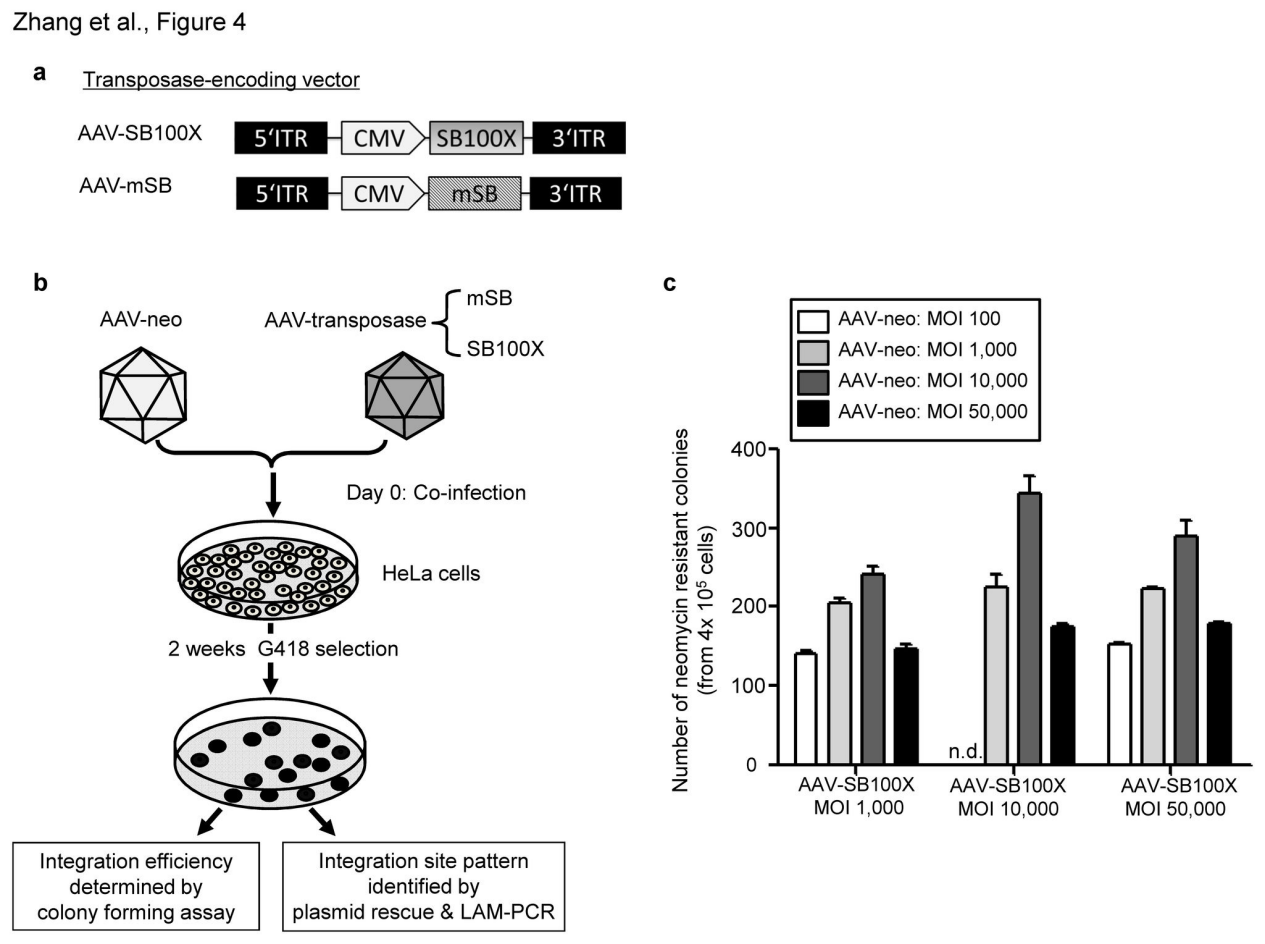
Transposition efficiency of the AAV/SB transposase hybrid-vector system. Colony forming assay to determine integration efficiencies from the hybrid AAV/SB vector system in HeLa-cells. HeLa-cells were co-infected with AAV-neo and AAV-SB100X at increasing dosages (MOI 100, 1,000, 10,000 and 50,000). (**a**) The AAV vector AAV-SB100X contains a transgene expression cassette for the hyperactive Sleeping Beauty (SB) transposases SB100X expressed under the control of the cytomegalovirus promoter (CMV). The control vector AAV-mSB encodes the mutated and inactive version of SB (mSB). (**b**) After co-transduction and selection, obtained cell colonies were either collected as pools for integration site analyses or colonies were stained with methylene blue and counted to determine integration efficiencies. (**c**) Result of the colony forming assay. The Y-axis shows the number of neomycin-resistant colonies obtained in different experimental settings. Error bars indicate standard deviation (n=3). n.d.: not determined.

The transposon-donor vector AAV-neo and the vector AAV-SB100X encoding hyperactive SB transposase under the control of the cytomegalovirus (CMV) promoter were used to evaluate transposition efficiencies (Figure **4a**). For achieving somatic integration mediated by SB transposase from the transposon-donor vector AAV-neo, it is mandatory that cells are co-infected with both vectors (Figure **1**). To optimize conditions for the AAV/SB transposase hybrid-vector system, we co-infected HeLa-cells with viral vectors AAV-neo and AAV-SB100X using varying MOIs of AAV-neo (MOI 100; MOI 1,000; MOI 10,000 and MOI 50,000) and AAV-SB100X (MOI 1,000; MOI 10,000 and MOI 50,000). Colony forming assays were performed and stable cell clones were quantified and analyzed on a molecular level with respect to vector genome copy numbers and integration sites within the host genome. A schematic outline of the procedure is shown in Figure **4b**. Highest numbers of colonies were obtained after co-infection with AAV-neo at MOI 10,000 and AAV-SB100X at MOI 10,000 (Figure **4c**). As a control, other groups received the control vector AAV-mSB expressing an inactive version of the SB transposase (mSB) (Figure **4a**) at varying MOIs (MOI 1,000; MOI 10,000 and MOI 50,000) along with the vector AAV-neo at MOI 1,000 and MOI 10,000. This experimental setup resulted in more than 10-fold decreased numbers of resistant cell clones in all mSB control groups (data not shown).

### Molecular analysis of transposition events

**Figure 5:**
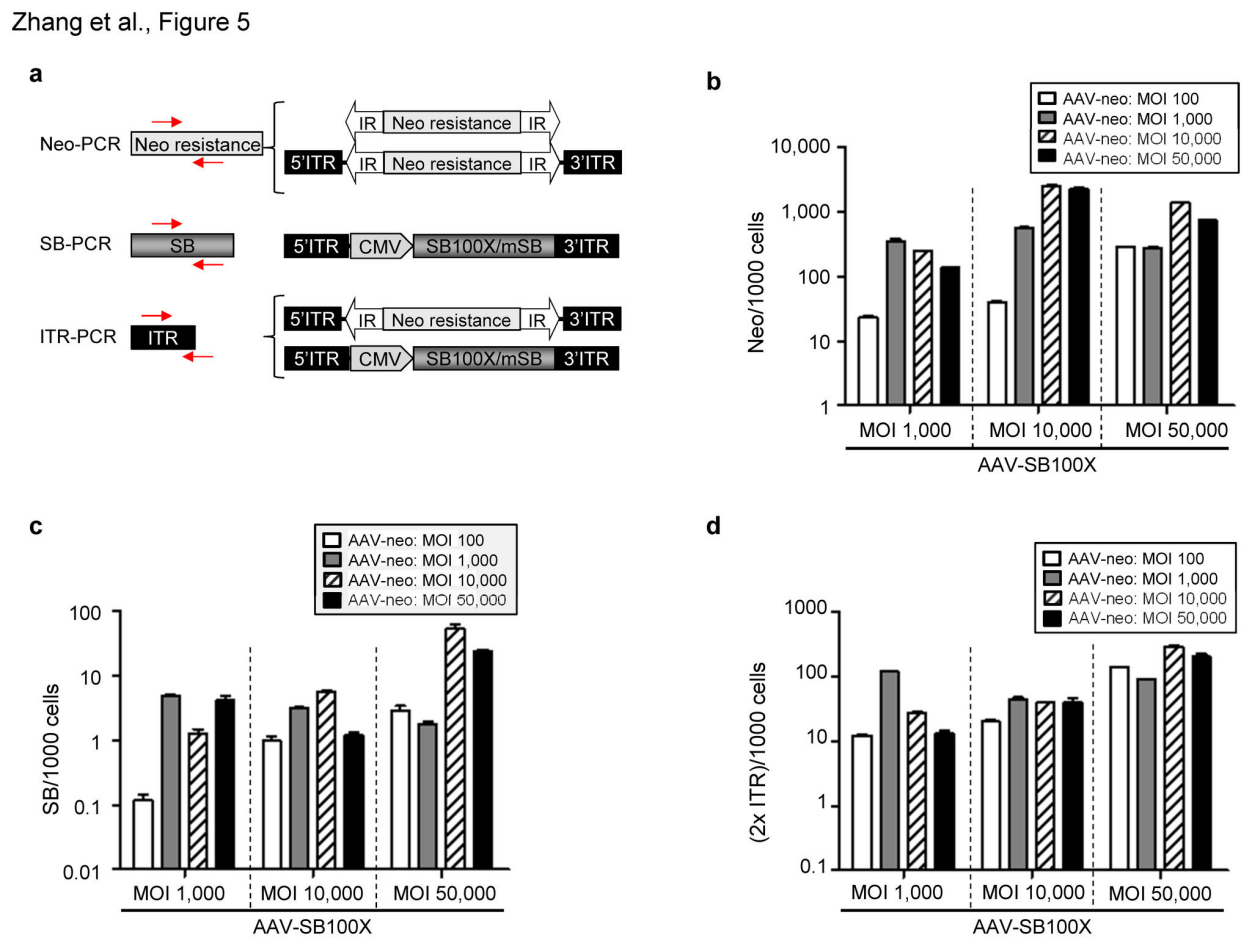
Genome copy numbers of each hybrid-vector element after AAV transduction. Genomic DNA of neomycin-resistant cells (from Figure 4) was analysed by quantitative real-time PCR (qRT-PCR) using primers and probe detecting AAV-ITRs and primers detecting the cDNAs of the two main functional genes neo (neomycin resistance gene) and SB (Sleeping Beauty transposase encoding gene). As internal control a PCR detecting hB2M (Beta-2-microgloblin gene) was performed. (**a**) PCR setup and location of primers used for quantitative analysis of vector genome copy numbers. The neo-PCR detects a 337 bp region in the neomycin resistance gene, which include integrated transposon, non- integrated and integrated AAV genomes. The SB-PCR detects an 82 bp region contained in the SB transposase encoding gene. Primers and probe specifically detecting the AAV ITR region [44] were used for amplification and detection of a 62 bp PCR product. Red arrows depict real-time PCR primer binding sites. (**b**) Neomycin copy numbers per 1000 cells after AAV transduction. Error bars indicate standard deviation (n=3). (**c**) SB transposase copy numbers per 1000 cells. Error bars indicate standard deviation (n=3). (**d**) 2XITR copy numbers per 1000 cells in co-transduced cells. Error bars indicate standard deviation (n=3).

To perform molecular analysis of AAV transduced cells, we determined DNA copy numbers of several components essential for the AAV/transposase hybrid-vector system by quantitative real-time-PCR (qRT-PCR). This PCR analysis included quantification of the neomycin resistance gene, the AAV ITR and the transposase encoding DNA (Figure **5a**). The Neo-PCR (Figure **5a**, upper panel) detected the neomycin encoding gene which included three potential molecular forms, comprising the transposon that is derived from SB-mediated integrations, the transposon derived from the original AAV-neo viral vector genome integrated into the host genome by AAV-vector mediated integration, and potential remaining episomal AAV vector genomes carrying the neo transgene. We detected >1 copies per cell of the neomycin gene in four groups which received a high dose of the AAV-neo vector (MOIs 10,000 and 50,000) along with a high dose of the vector AAV-SB100X (MOIs 10,000 and 50,000) (Figure **5b**). Notably, highest neomycin copy numbers (2,500 neomycin copies/1,000 cells) were detected in cells which were co-infected with both vectors at MOI 10,000 (Figure **5b**). The SB-PCR (Figure **5a**, middle panel) detects the SB transposase genes (SB100X and mSB). These stably maintained SB encoding DNAs are most likely due to AAV-vector mediated integration of AAV vectors AAV-SB100X or AAV-mSB. The SB transposase copy numbers revealed that less than <1 copy per cell (10 SB copies/1000 cells) were maintained in most groups, except for the two groups which received a high dose of AAV-neo (MOI 10,000 and 50,000) and MOI 50,000 of AAV-SB100X (Figure **5c**). The AAV-ITR PCR measures total AAV viral genome copy numbers directly matching the number of AAV vector DNA molecules in transduced cells (Figure **5a**, lower panel). We detected AAV-SB100X dose-dependent effects on ITR copy numbers in cells which were co-infected with AAV-neo at MOI 100, MOI 10,000, MOI 50,000 (Figure **5d**).

**Figure 6:**
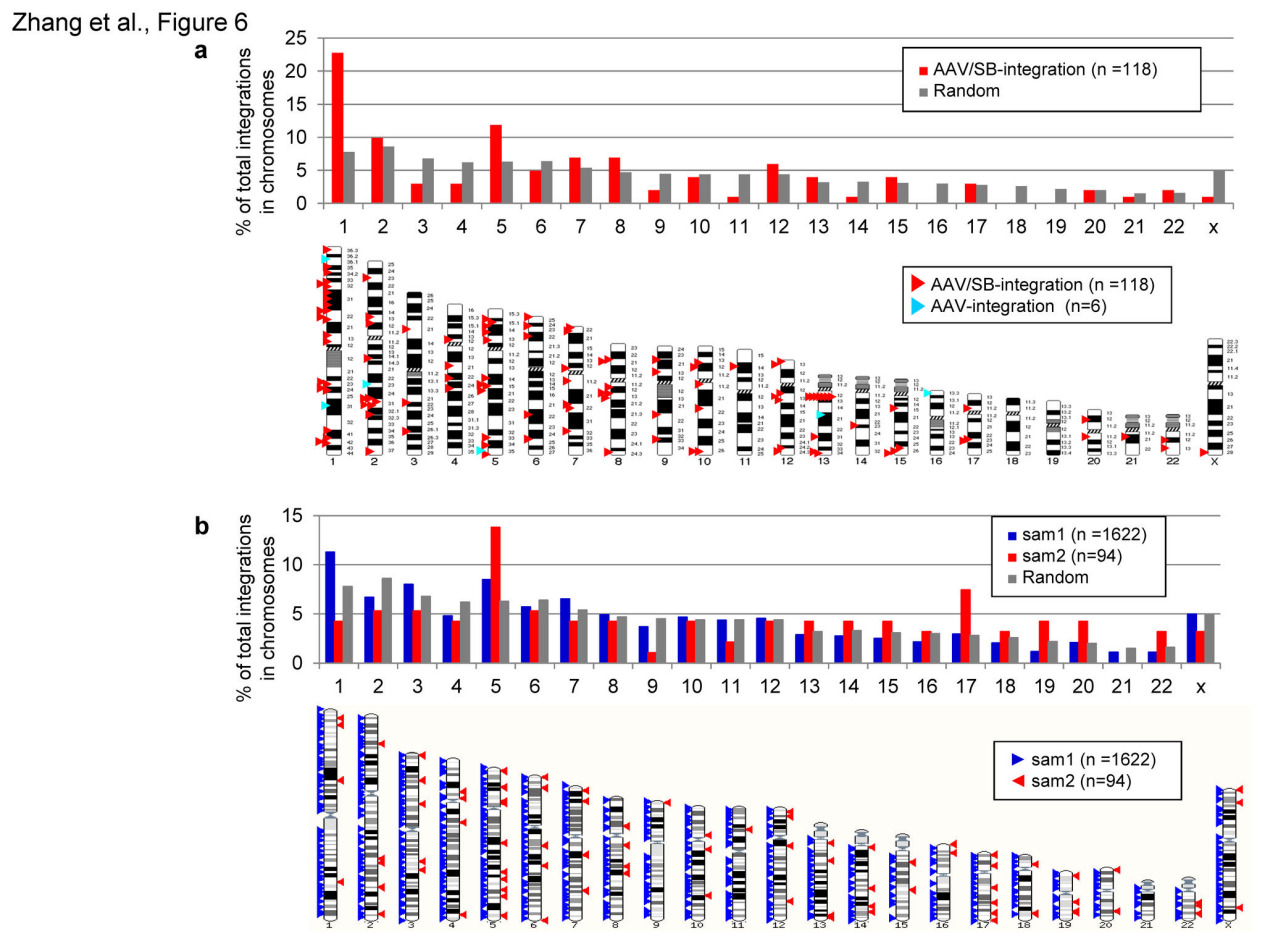
Integration sites from the hybrid AAV/SB vector system identified by plasmid rescue and LAM-PCR approaches. (**a**) Integration events identified by plasmid rescue. Percentages of integration events within each individual chromosome in comparison to a random control set is depicted in the upper panel. A schematic overview of chromosomal distribution is shown in the lower panel. The relative position of all unique integration sites identified from AAV/SB hybrid vector system infected cells were mapped within the human genome. Respective triangles indicate the relative positions of the chromosomal integration site observed (red triangle for SB-mediated integration; blue triangles for AAV -mediated integration). (**b**) Sequence reads and chromosomal distribution identified after performing LAM-PCR in AAV/SB hybrid-vector infected HeLa-cells (upper panel). Integration sites were compared to a computer-simulated random integration profile [13]. The lower panel shows mapping of all 1716 unique integration sites identified by LAM-PCR. Respective triangles indicate the relative positions of the chromosomal transposon integration site observed from sample 1 (blue triangles, AAV-neo MOI 10,000 and AAV-SB100X MOI 10,000) and sample 2 (red triangles, AAV-neo MOI 10,000 and AAV-SB100X MOI 50,000).

Next we wanted to analyze the integration profile of our AAV/transposase hybrid-vector system. As controls for integration site analysis in the absence of transposase, we performed colony forming assays utilizing the inactive and mutated version of SB transposase (mSB). However, we did not perform colony forming assays for all possible vector combinations and ratios as shown in Figure **4c**. We only analyzed the AAV-neo vector at MOI 1,000 and MOI 10,000 in combination with the AAV-mSB vector at MOI 1,000 and MOI 10,000, and MOI 50,000 (Table **S2**), because these MOIs also gave the best results when using the hyperactive Sleeping Beauty transposase SB100X (Figure **4c**). For detailed characterization of transposition events on a molecular level, we identified integration sites using a strategy based on plasmid rescue (Figure **6a**) and a linear amplification-mediated PCR (LAM-PCR) protocol (Figure **6b**). The plasmid rescue method allows differentiating between actual transposition events from the AAV vector genome and integration of the complete AAV genome due to AAV-vector mediated integration because sequences flanking both ends of the AAV vector genome can be determined. If AAV-vector mediated integration occurs, rescued AAV-vector mediated integration events should still carry the AAV-derived ITRs and the inverted repeats (IRs) from the transposon, while rescued transposition events should solely contain SB transposon IRs. Towards this end we analyzed genomic DNA from cells which were co-infected with AAV-neo and AAV-SB100X at MOI 10,000 for both recombinant viruses. We isolated a total of 99 integration events of which 96 events were identified as transposition events and three events were AAV-vector mediated integration events (Table **1**). From the other groups which received different MOIs, we rescued 13 transposition events. For the mSB group 7 integration events were identified of which four were AAV-vector mediated integration events (Table **1** and Table **S2**). Compared to a random chromosomal distribution, chromosomal distribution of transposition events after infection with the AAV/transposase hybrid-vector system revealed a slight bias towards transposition into chromosomes 1 and 5 (Figure **6a**). Using the LAM-PCR method we identified a total of 1716 transposition events (1622 for sample 1 and 94 for sample 2) which were characterized on a molecular level. In concordance with the plasmid rescue protocol we observed a slight integration bias towards chromosome 1 and 5 for sample 1 (AAV-neo MOI 10,000 and AAV-SB100X MOI 10,000) in the LAM-PCR analysis. For the second sample (AAV-neo MOI 10,000 and AAV-SB100X MOI 50,000) there was a slight bias towards integration into chromosomes 5 and 17. A summary of all integration sites obtained by plasmid rescue and LAM-PCR is provided in Table **1** and Figure **6** **.**

**Table 1:** AAV- and SB-mediated integrations and number of rescued plasmids from each group.

**AAV-neo**	**MOI 100**			**MOI 1,000**			**MOI 10,000**			**MOI 50,000**		
	% SB	IS PR	IS LP	% SB	IS PR	IS LP	% SB	IS PR	IS LP	% SB	IS PR	IS LP
**AAV-SB100X**												
MOI 1,000	47.4	0/0	n.d.	66.0	1/0	n.d.	88.6	6/0	n.d.	90.4	0/0	n.d.
MOI 10,000	50.3	0/0	n.d.	92.1	2/0	n.d.	98.5	96/3	1622	98.2	2/0	94
MOI 50,000	51.4	2/0	n.d.	66.8	0/0	n.d.	78.7	0/0	n.d.	72.7	0/0	n.d.
**AAV-mSB**												
MOI 1,000	n.p.	n.p.			0/0	n.d.		0/2	n.d.	n.p.	n.p.	
MOI 10,000	n.p.	n.p.			1/1	n.d.		2/0	n.d.	n.p.	n.p.	
MOI 50,000	n.p.	n.p.			0/1	n.d.		0/0	n.d.	n.p.	n.p.	

% SB: provides the percentage of SB transposase-mediated integration events in all analysed integration sites calculated by the gene copy number measured by quantitative real-time PCR (see also Figure 8). IS PR: provides the number of rescued transposition events and the AAV-vector mediated integration events (No. of transposition events/ No. of AAV vector integration events). IS LP: provides the number of rescued transposition events using LAM-PCR. Vector combinations which were not performed are noted as n.p. 0/0: >20 colonies were picked for analyses but no transposition events and/or AAV-vector integration events were rescued. n.d.: not determined.

**Figure 7:**
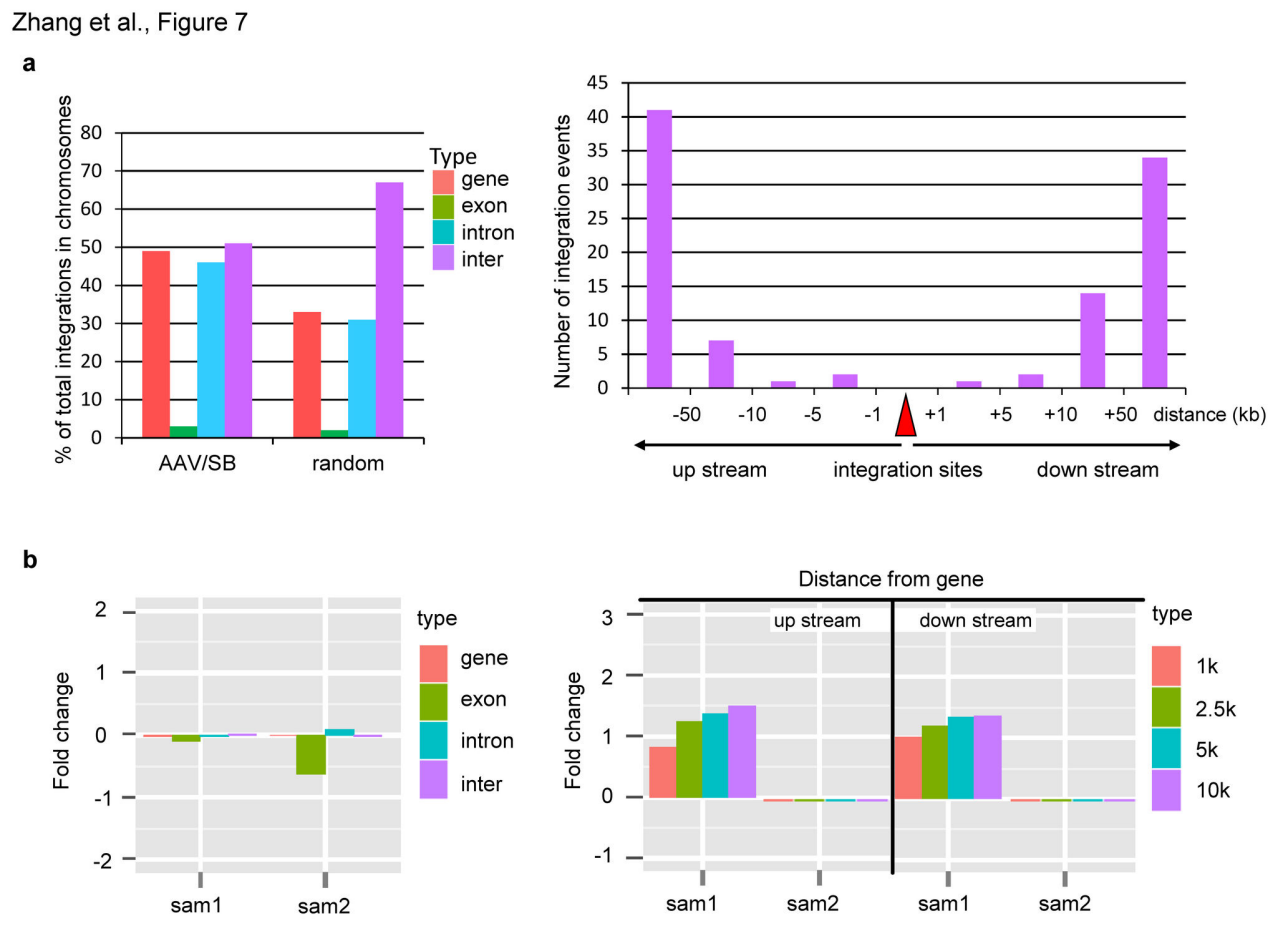
Frequencies of insertions from hybrid AAV/SB vector system within or outside of genes. (**a**) Characterization of AAV/transposase hybrid-vectors mediated integrations identified by plasmid rescue. Percentage of integration sites which were identified to be intragenic (in introns, or in exons) and intergenic after performing plasmid rescue (left panel). Random: computer predicted data [13]. The right panel shows the distance to the nearest genes of integration sites which hit intergenic regions. Distances of genes upstream and downstream of the transposition event are depicted. (**b**) Characterization of AAV/transposase hybrid-vectors mediated integrations identified by LAM-PCR method. Insertion frequencies compared to a random dataset are shown with respect to integration within and outside of RefSeq genes (left panel), and distances upstream and downstream of genes (right panel). The bars depict fold changes of integration frequencies compared to the random distribution profile. Abbreviation: sample 1 (AAV-neo MOI 10,000 and AAV-SB100X MOI 10,000) and sample 2 (AAV-neo MOI 10,000 and AAV-SB100X MOI 50,000).

To evaluate genotoxicity of the AAV/transposase hybrid-vector system we analyzed whether transposons landed in gene or non-gene areas. Interestingly, for the plasmid rescue method 49% percentage of integration sites were identified to be intragenic (Figure **7a**, left panel). Among these intragenic integrations only 3 were in exons and the remaining transposition events were found to be located in introns (53, 95%). Most intergenic integrations were found to be far away from genes (94% locate >10 kb away) (Figure **7a**, right panel). For the LAM-PCR method we observed a random integration pattern with respect to integration into intra- and intergenic regions (Figure **7b**, left panel). Also, the distance of integration in close proximity to genes was not changed significantly compared to a random integration profile (Figure **7b**, right panel).

**Figure 8:**
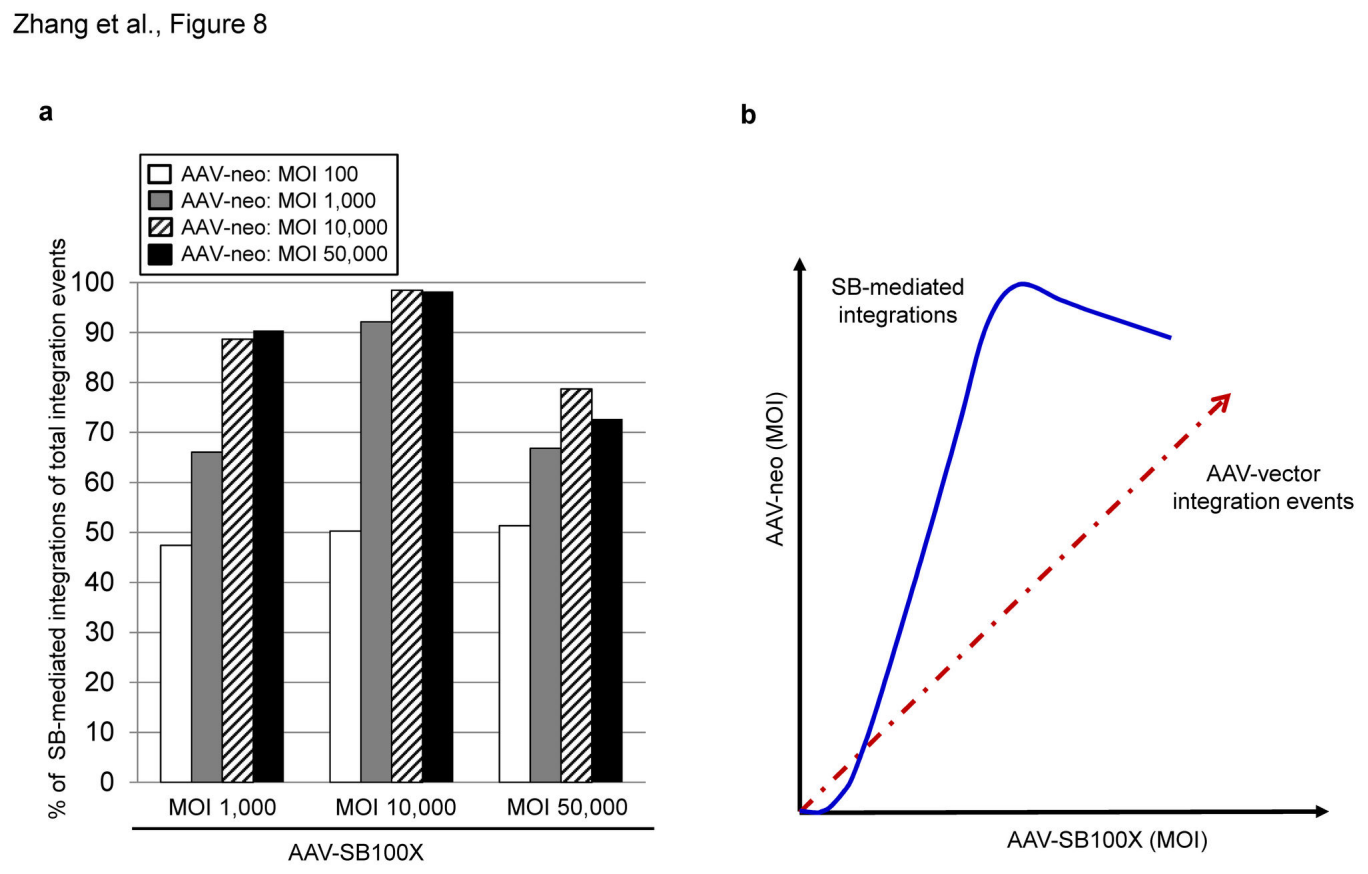
Transposition efficiencies are dose-dependent. (**a**) To calculate the ratio of SB transposase-mediated integration versus AAV-vector mediated integration, the percentage of SB-mediated integration events contained in all integration events (SB-mediated integration events + AAV-vector mediated integration events) was determined. Calculation: (neomycin gene copy number determined by qRT-PCR) – [(AAV-ITR copy number determined by qRT-PCR) – (SB copy number determined by qRT-PCR)] / (neomycin gene copy number determined by qRT-PCR) x 100. It is of note that the low percentage of potentially remaining non-integrated AAV vector genomes should also be detected by the neomycin-specific primers. (**b**) Schematic overview of dose-dependent transposition efficiencies. The X-axis indicates increased dosages of the transposase encoding vector AAV-SB100X while the Y-axis indicates the increase of the AAV-neo vector. The dashed red arrow represents the trend of AAV-vector mediated integrations and the blue curve displays the efficiency of SB-mediated integrations in a dose-dependent manner.

To estimate to which extent SB transposase-mediated transposition contributes to stabilization of the transgene after transduction of cells with AAV vectors, we performed a calculation based on the qRT-PCR data (Figure **5**). We found that the percentage of SB-mediated integration depends on the MOI of both transposase and transposon-donor vectors (Figure **8a**). In those groups which received the lowest dose of the vector AAV-neo (MOI 100), SB-mediated integration events corresponded only to 50% of all integration events containing the neomycin transgene. For the group which received AAV-neo and AAV-SB100X at MOIs 10,000, showing highest numbers of colonies in the colony forming assay (Figure **4c**), 98% of all stabilized neomycin transgene copies were from SB-mediated integrations. The vector dose-effect on transposition efficiencies and potential AAV-vector mediated integrations from the AAV/transposase hybrid-vector is schematically shown in Figure **8b**.

## Discussion

AAV vectors are broadly used in clinical applications and therefore this type of vector holds great promise for gene therapeutic applications [2,4]. However, in cycling and regenerating tissues the genetic payload gets lost rapidly [7] because of the lack of a stabilizing genetic element during mitosis. Herein, we developed the first prototype of a recombinant AAV vector which utilizes the SB transposase somatic integration machinery for stabilized transgene expression in combination with the high transduction efficiencies of AAV.

We first assessed whether excision of the transposon from the various molecular forms of the AAV vector genome [16] after cellular transduction is required. We found that Flpe mediated excision and circularization of the transposon from the AAV vector genomes is not mandatory (Figures **S2** and **S3**). After AAV transduction circular monomers and concatemers and linear integrated and non-integrated vector DNA molecules are produced [16] and these seem sufficient to serve as substrates for transposition (Figure **1**). In fact, Flpe recombination seems to decrease transposition efficiencies in a plasmid based context (Figure **S2b**) which may be caused by the fact that Flpe recombination [17] represents a bidirectional reaction. FRT recombination events between integrated and episomal AAV vector forms may occur which could interfere with the transposition reaction. This finding is important for the molecular design of the AAV/transposase hybrid-vector system, because FRT sites and Flpe encoding sequences can be deleted from the AAV-hybrid vector system, significantly simplifying the molecular setup. This feature of the AAV/transposase hybrid-vector system may be advantageous compared to the previously published adenovirus/transposase hybrid-vector system [9,18] for which Flpe mediated excision of the transposon from the adenovirus vector genome is required. Notably, Flpe expression seems to have different effects on colony forming numbers dependent on whether the Flpe transgene was introduced into target cells by plasmid transfection or by an adenoviral vector infection (Figure **S2** and Figure **S3**). However, in contrast to experiments shown in Figure **S2**, the adenovirus experiments were performed in the stably expressing SB100X cell line which means that SB100X was already expressed before Flpe was introduced. As a consequence Flpe may have less pronounced effects on the outcome of the experiment. As a second point, it should be mentioned that in contrast to plasmid transfection, adenovirus infection results in significantly decreased copy numbers of the Flpe transgene and its translated protein. This in turn may lead to enhanced Flpe effects in cells which received the Flpe transgene based on plasmid transfection.

We tested and compared two different hyperactive SB transposase versions (HSB5 and SB100X) and found that in the context of an AAV vector SB100X and HSB5 showed 10-fold and 2-fold increased activity compared to the mSB control group (Figure **3**). Therefore, we pursued our further experiments with the hyperactive Sleeping Beauty transposase SB100X. A previously published lentivirus-transposase hybrid-vector utilized hyperactive SB transposases SB100X, SB80X and HSB3 (a progenitor of HSB5) for somatic integration from an integrase-deficient lentiviral vector (IDLV) [19]. Also in this study SB100X but also SB80X were superior to HSB3 with respect to transposition efficiencies. Another study applied hyperactive SB transposase SB11 displaying 3-fold increased transposition activity compared to wild type SB transposase (SB10), but the respective transposition efficiencies not compared to other hyperactive or inactive SB transposase versions [15]. However, in contrast to studies using IDLV for transposon delivery, AAV vector integration itself is unavoidable for our AAV/transposase hybrid-vector. However, somatic integration efficiencies of recombinant AAV vectors are rather low [7], which is also in concordance with the findings of the present study.

In contrast to experiments involving AAV transduction (Figure **2** and Figure **3**), we observed approximately 100-fold increased transposition activities when using plasmids as transposon-donor and transposase encoding vector (Figure **2** and Figure **S2**). However, for plasmids transfection efficiencies are usually high and hundreds of plasmid copies will co-transfect target cells. In contrast, if one component of the SB transposase system or both transposon-donor and transposase encoding vector are delivered by an AAV vector, transduction efficiencies are significantly lower compared to plasmid transfection. This is mainly due to the fact that in general AAV transduction efficiencies in immortalized cells in vitro are lower than with plasmid transfection. One could also speculate that plasmids entering the target cell already as a circular DNA genome are more efficient in serving as substrates for transposition than AAV vectors which enter the cell as single-stranded DNA genomes which are subsequently converted into double-stranded DNA. This was also the reason for generating stably transposase-expressing cell lines in our initial study (Figure **2a and b**), because this strategy would bypass second-strand synthesis and the transcription and translation steps of the transposase. Although it seems that for the used cell clones (clone 3 for SB100X and clone 3 mSB) there are higher expression levels of SB100X compared to mSB (Figure **2b**), it is of note that SB100X and mSB transposase transcription was detected by regular reverse transcription PCR and that we did not quantify transcription levels. Nevertheless, in the stable cell lines it may also be possible that there are different integrated vector genome copy numbers of SB100X and mSB transgenes inserted into the host genome and thereby also different expression levels. However, we believe that this should not significantly influence final transposition efficiencies. If there is more mSB, anyhow the mSB is inactive for transposition. In case there is too much SB100X expressed in this cell line, we believe that it is unlikely it may increase transposition activities. The main effect, overexpression of SB100X may have on the cells, may be over-production inhibition or SB transposase mediated cytotoxicity. It is of note that not only the vector genome copy numbers of the integrated plasmid may influence SB100X and mSB expression levels, but also the locus in the host chromosomes into which the plasmid integrated.

With respect to the integration profile analyzed by LAM-PCR and plasmid rescue we found a close to random integration pattern with a moderate bias towards integration into chromosome 5 for both methods and all analyzed samples (Figure **6**). Therefore, we concluded that the integration pattern mainly mimicked transposition events observed in non-viral approaches and for the IDLV/SB transposase hybrid-vector system [13-15,19]. Slightly enhanced integration into chromosome 1 (Figure **6**) may be explained by the fact that chromosome 1 represents the largest chromosome in the human genome. In addition, it could be speculated that analyzing more integration sites may decrease the marginal bias towards certain chromosomes which was also observed in other studies [13-15,19]. With respect to integration into genes and non-gene areas we found that for the plasmid rescue method up to 49% of the integration events landed in genes and for the LAM-PCR we observed a close to random distribution of transposition events (Figure **7**). Compared to other studies this transposon integration profile is similar to the one observed in other studies for which SB transposase-mediated integration efficiencies into genes varied form 39-53% [13-15,19]. It is of note that for conventionally used recombinant AAV vectors integration efficiencies into gene regions range from 39% [20] to 53% [21] in vitro and reach up to 61% in vivo [21].

We analyzed the relation between transgene stabilization and AAV vector dosages used for cell transduction. Determined genome copy numbers of the transposon encoding neomycin, the SB transposase and the AAV-ITR, together with the identified integrations gave basic information about the AAV-transposase hybrid-vector system. We found that transgene stabilization due to SB transposase-mediated integration was vector dose-dependent (Figures **4**, **5**, **8** and Table **1**). As shown in Figure **5b**, in four groups which received high vector dosages (MOIs 10,000 and 50,000), >1 copy/cell of the neomycin gene was detected indicating that more than a single integration event occurred per single cell. Moreover, we observed a relatively high amount of transposase genome copy numbers compared to the mSB control group (Figure **5c** and Table **S2**). At the time we can only speculate about the reasons responsible for this phenomenon. We used a high dose of the AAV-SB100X vector and since one of the intrinsic properties of SB transposase is introducing double-strand breaks into the genome, random integration frequencies may be increased. Another possibility could be that the transposase encoding sequence retained some residual transposon activity which could result in increased somatic integration in groups which received the hyperactive transposase SB100X. A previous study by Huang and colleagues [22] also detected high copy numbers of integrated transposase transgenes in transduced cells. However, compared to our study integration efficiencies in the presence of inactive SB transposase proteins were not analyzed. With respect to safety, somatic integration and subsequent long-term SB transposase expression in transduced cells may harbor the risk for unwanted side effects such as secondary transposition. However, the work by Huang and colleagues and another study by Bell and colleagues [23] indicated that the maintained SB transposase encoding transgenes are silenced over time, lowering the risk of unwanted side effects.

We found that AAV-vector mediated integration occurs not only in the mSB groups but also in the groups which received SB100X (Table **1**). As AAV seems to have a strong preference for integrating near gene regulatory sequences and transcriptional start sites [21], it is important to study how to control and restrain AAV-vector mediated integrations while maintaining high transduction and transposition efficiencies. Towards this end, we aimed at analyzing SB-mediated transposition efficiencies in relation to AAV vector integration which is schematically shown in Figure **8**. Notably, when using an MOI of 50,000 for both vectors, we observed a decrease of transposition efficiencies which may be due to an over-production effect [24,25]. Furthermore, increasing the MOI of AAV-SB100X led to decreased numbers of colony forming units compared to the MOI 10,000 to MOI 10,000 ratio of AAV-neo and AAV-SB100X, respectively (Figure **4**). The effect may be due to the fact that an increased number of AAV vector genomes encoding SB100X may integrate into the host genome potentially resulting into toxicity due to stable expression of the hyperactive transposase gene.

Another interesting finding can be derived from the groups which received the transposon-donor vector (AAV-neo) and the inactive version of transposase mSB (AAV-mSB). In contrast to the active transposase groups, genome copies of neomycin and AAV-ITR sequence copy numbers showed relatively high levels (Table **S2**). For instance, a similar level of neomycin and AAV-ITR copy numbers (~ 300 copies/1000 cells) was detected in the group which received AAV-mSB at MOI 50,000 and AAV-neo at MOI 1,000. This underlines that integration events are mainly AAV-vector derived. However, we identified a very limited number of SB-mediated integrations from groups which received mSB (Table **1**), which may be explainable by the fact that mSB displays residual transposase activity.

Our AAV/transposase hybrid-vector system represents the first prototype of an AAV vector capable of maintaining transgene expression even during rapid cell cycling. Hybrid-vector technologies were also used for adenoviral vectors, herpes-simplex virus based vectors and the integrase-deficient non-integrating lentivirus technology [26-32]. For these hybrid-vector systems various genetic elements for retention and replication of the therapeutic DNA after cellular transduction were evaluated. These genetic elements included bacteriophage derived integrase PhiC31 for somatic integration into genomic hot spots [33], zinc finger nucleases (ZFNs) and transcription activator like elements (TALEN) [34-36] for gene insertion at pre-determined genomic loci, retrotransposons from somatic integration [37], AAV Rep protein for site-specific integration in chromosome 19 [38], and genetic elements such as S/MAR based vectors [39] and Epstein-Barr virus based vectors [40,41] for episomal maintenance of the transgene. In the future, AAV may also be combined with other genetic elements for stabilized transgenes expression besides the SB transposase system.

In summary, we show for the first time that AAV vectors can serve as template for SB transposase-mediated somatic integration. With further improvements regarding SB transposase-mediated integration efficiencies from the AAV vector genome and AAV-transduction efficiencies, this vector system can pave the way towards treatment of diseases which require stable gene transfer into rapidly dividing cells.

## Materials and Methods

### Cell lines

HeLa-cells (human cervical cancer cells) and human embryonic kidney cells (HEK293-cells) were cultured in Dulbecco’s modified Eagle’s medium (DMEM, PAA) supplemented with 10% fetal calf serum. All cell lines were cultured at 37 °C in a 5% humidified atmosphere.

Transposase stably expressing HEK293-cells were generated by stably transfecting plasmids pIRES-Puro-SB100X or pIRES-Puro-mSB. Single cell clones were amplified under puromycin selection pressure and tested for SB transposase expression using reverse-transcription PCR.

### Colony forming assays

For colony forming assays solely based on plasmid transfection, HeLa-cells were seeded in 6-well plates at a density of 3 × 10^5^ cells/well in 3 ml medium. Transfection was carried out the next day in serum-free medium with FuGENE 6 (Promega) according to manufacturer’s protocol. A total of 2 µg of plasmid DNA with molar ratio of 1:2 between transposase–encoding plasmid and transposon substrate plasmid was transfected using a FuGENE-to-plasmid ratio of 3:2. After 2 days, the transfected cells were trypsinized and reseeded in 10-cm dishes at different dilutions. Two days later the cells were selected in Geneticin-containing medium (500 µg/ml G418) for 14 days. Drug-resistant colonies were either stained with methylene blue (Sigma-Aldrich) and counted or collected as cell pools for integration site analysis.

For colony forming assays based on plasmid transfection and subsequent AAV infection, HeLa-cells cultured in 6-well plates were first transfected with 1µg (100,000 copies per cell) of the respective transposase encoding plasmid (pCMV-mSB, pCMV-HSB5, or pCMV-SB100X). One day post-transfection cells were infected with the vector AAV-neo at MOI 10,000. Two days post-infection cells were harvested and seeded using cell densities of 4x10^5^ cells per 10-cm dish. After 14 days under selection pressure, cell colonies were either collected as pools for integration site analysis or the colonies were stained with methylene blue and counted to determine transposition efficiencies.

When performing colony forming assays relying on co-infection with the transposase encoding vector AAV-SB100X and the transposon-donor vector AAV-neo, HeLa-cells at 50-80% confluency grown in 6-well plates were co-infected with AAV-neo and AAV-SB100X at increasing dosages (MOI 100, 1,000, 10,000 and 50,000). Two days post-infection cells were harvested and seeded at cell densities of 4x10^5^ cells per 10-cm dish. After 14 days under selection pressure, surviving cells were either collected as pools for integration site analysis or the colonies were stained with methylene blue and counted to determine transposition efficiencies.

All plasmid transfection and viral vector infection were performed in triplicate.

### Cloning of recombinant AAV vectors

To clone the AAV production plasmid pAAV-neo, the plasmid pZac2.1 (obtained from Jim Wilson, University of Pennsylvania, Philadelphia, PA, USA) containing the AAV ITRs was cut with the restriction enzyme *Sna*BI and the blunted I-*Ceu*I/PI-*Sce*I fragment from pHM5-attB-T_MCS_-FRT2 [18] was ligated into that side resulting into the plasmid pZAC- attB-T_MCS_-FRT2. To clone the AAV production plasmid pAAV-neo, we cloned the *Eco*RI fragment from the plasmid p11 with the neomycin resistance gene [42] into the *Pme*I site of pZAC- attB-T_MCS_-FRT2.

Plasmids pCMV-HSB5, pCMV-mSB and pCMV-SB100X and the FLPe encoding plasmids used as PCR templates were published previously [9,10,33] To clone AAV production plasmids pZIF-SB100X-Flp, pZIF-HSB5-Flp, pZIF-mSB-Flp we PCR amplified the IRES sequence (619 bp) from the pIRESpuro vector (Clontech) using primers IRES-forw-XbaI/BclI and pIRES-rev- XbaI/BamHI. Furthermore, we amplified the Flpe gene (1300 bp) using primers Flpe-forw-BamHI and Flpe-rev-NotI and the SB transposase gene (1022 bp) using template plasmids pCMV-HSB5, pCMV-mSB and pCMV-SB100X and primers SB-forw-XhoI and SB-rev-EcoRI. Resulting PCR products were cloned into the respective sites of the AAV cloning vector pZac2.1. To clone vectors pZAC-SB100X, pZAC-HSB5 and pZAC-mSB DNA sequences were PCR amplified using the same primers and cloned into the XhoI and EcoRI sites of the plasmid pZac2.1. For detailed description of each primer pair please refer to Table **S1**.

### AAV production and titration

Recombinant AAV vectors were generated utilizing calcium phosphate mediated triple transfection of HEK293-cells [43]. Recombinant AAV vector genomes were packaged into capsids from AAV-2 using the helper plasmid pRC (gift from Hildegard Büning, University of Cologne, Cologne, Germany) and the adenoviral helper plasmid pAdDeltaF6 (obtained from Jim Wilson, University of Pennsylvania, Philadelphia, PA, USA). For final AAV production, plasmids containing the gene of interest need to be packaged into AAV virions, we used plasmid pAAV-neo (containing the neomycin resistance gene) and plasmids pZAC-SB100X and pZAC-mSB (containing transgene expression cassettes for the hyperactive Sleeping Beauty transposase SB100X and the mutated and inactive version mSB, respectively). For triple-transfection of the two helper plasmids and the transgene encoding AAV vector genome, 35 µg of plasmid DNA (mixed in equimolar ratios) was used per 15 cm cell tissue culture plate. Three days after transfection, AAV vectors were harvested. For purification, the ViraBind™ AAV Purification Mega Kit (Cell Biolabs) was used according to the manufacturer’s instructions.

### SB100X and mSB reverse transcription PCR to analyse expression in stably transduced cells

The RNA of 3.2x10^6^ cells was isolated using the RNeasy kit (Qiagen) following the manufacturer instructions. After determining the RNA amount by measuring the optical density, 1 µg RNA was used for reverse transcription using polydT primer supplied in the First strand DNA synthesis kit (NEB). The generated cDNA was then subjected to a PCR reaction with SB100X and mSB specific primers SB100-for and SB100-rev (Table **S1**).

### Quantification of vector genome copies by quantitative real-time PCR (qRT-PCR)

For the quantification of the major genes (neo and SB) from each vector, qRT-PCR with the primer pairs specific for the respective gene sequences were performed using the CFX96 Touch™ Real-Time PCR Detection System (Bio-Rad). The PCR was carried out with the following program: pre-incubation/activation at 95°C for 5 min, amplification and data collection during 40 cycles (95°C for 15 s and 60°C for 1 min), and subsequent melt curve analysis from 55 to 95 °C. iQ™ SYBR® Green Supermix (Bio-Rad) was used for the PCR setup.

A primer pair and probe [44] binding to the ITR sequence of AAV was used to determine the copy number of AAV vector genomes in transduced cells. The PCR was based on the following program: pre-incubation/activation at 95°C for 5 min, amplification and data collection during 40 cycles (95°C for 15 s and 60°C for 30 s). The Sso Fast™ Probes Supermix (Bio-Rad) was used for these PCRs.

To normalize the different samples, the same amount of genomic DNA was analyzed by real-time PCR detecting the human B2M gene (Beta-2-microgloblin). For detailed description of each primer pair please refer to Table **S1**.

In the quantitative PCR all standards and samples were performed in triplicate.

### Mapping of integration sites

Integration sites were rescued from AAV/SB-transduced cells by plasmid rescue method as previously described [42]. Briefly, genomic DNA isolated from selected geneticin-resistant cell clones was first digested with restriction enzymes *Nhe*I, *Spe*I and *Xba*I producing compatible ends (5’ CTAG). Importantly, these restriction enzyme recognition sites are not present in the integrated transgene expression cassette. The genomic DNA fragments were subsequently self-ligated to generate plasmids, which contain a neomycin resistance transgene and a bacterial origin of replication, which are both included in the transposon, and the respective genomic DNA fragment of the integration sites. After transformation into DH10B competent cells, single bacterial colonies were amplified and respective DNA of plasmids was purified. These plasmids were pre-screened by *Hind*III restricted enzyme, which releases a 2.5-kb band from the integrated transgene. Only plasmids contained the 2.5 kb fragment after *Hind*III digest and a unique DNA segment larger than 200 bp were sequenced utilizing the primers binding to the left IR (L-IR) or the right IR (R-IR). Sequences flanking the IRs contained genomic DNA sequences connected by the trail of the restriction enzymes (RE) at the end which were used for restriction enzyme digests of genomic DNA.

The generation of Sleeping Beauty transposase-mediated integration site libraries via LAM-PCR in combination with an Illumina Genome Analyzer platform was performed as described previously [14,19,45]. Briefly, three enzymes NlaIII (NEB), MluCI (NEB), FspBI (Fermentas) were used to prepare independent reactions for LAM-PCR.

### Bioinformatic analysis of integration sites

Sequences were blasted against the human genomic sequence database from NCBI (http://www.ncbi.nlm.nih.gov/genome/seq/BlastGen/BlastGen.cgi?taxid=9606). Integration events were considered authentic only if they (1) contained the sequence from the sequencing primer until the end of the inverted-repeat (IR), (2) matched a genomic location starting immediately after the end of IR (TCAACTG) and starting with a TA-dinucleotide sequence, (3) showed >95% identity to the genomic sequence, and (4) matched no more than one genomic locus with the highest identity. Only sequences combining all these four features were used for further analyses. For the identification of AAV vector-mediated integrations, the genomic locations need to start after the AAV ITR.

For LAM-PCR only the reads with exact adaptor sequence (23nt) were retained for subsequent analysis. The remaining part of the single reads containing putative genomic sequences were cut after 50nt. We mapped these reads to genome while only the sites with TA nucleotides were accepted. It is of note, that for the LAM-PCR dataset, AAV-vector mediated integration events were neglected. To map the individual integration events in human chromosomal DNA, the program Bowtie was used. To improve the data quality, we discarded all sequences for which we obtained <5 reads. To map these integrations identified by LAM-PCR back to individual chromosomal DNA, the Ensembl genome browser was used (the GRCh37.p10 Primary Assembly of the human genome). For statistical analysis, we used random control sets with 10,000 sites each as described previously [14,19,45].

### Statistical analysis

All experiments in this study were performed in triplicate. All data are reported as mean with standard deviation unless otherwise noted. Statistical comparison was made using the two- tailed student’s test, and a value of p < 0.05 was considered to be relevant compared to the respective control group.

## Supporting Information

Figure S1 **Molecular design of the AAV/SB hybrid vector system.** For somatic integration cells are simultaneously infected with the transposase-vector and the transposon donor vector. After entering the cell, AAV vector genomes form different molecular forms including circular monomers, dimers, and concatemers as well as linear monomers and concatemers. Two strategies were pursued to mobilize the transposon form the AAV vector representing the transposon-donor: (I) the transposon flanked by inverted repeats (IR, white horizontal arrows) is directly mobilized from the AAV vector genome by the SB transposase protein provided in trans, and integrated into a genomic target site (TA dinucleotide) (II). After entering the cell, Flpe recombination excises and circularizes the transposon from the various forms of the AAV vector genomes by recognizing the FRT sites contained in the transposon donor vector. Subsequently, the transposon flanked by IRs is mobilized from the circular intermediate by the SB transposase protein provided in trans. As a last step the transposon integrates in the host genome (waved black lines) into the genomic target site (TA-dinucleotide).
(TIFF)

Figure S2 **The effect of Flpe expression on transposition efficiencies.** Colony forming assays were performed in HeLa-cells. (**a**) The Sleeping Beauty transposase encoding AAV plasmids pAAV-SB100X, pAAV-SB100X-Flpe, express transposase under the control of the cytomegalovirus promoter (CMV). Plasmids pCMV-SB100X and pCMV-mSB also encode active and inactive SB transposase genes and the plasmid pCMV-Flpe expresses codon optimized Flp recombinase under the control of the CMV promoter. (**b**) Colony forming assays were performed in HeLa-cells. Equal molar ratios for plasmids pAAV-SB100X, pAAV-SB100X-Flpe, pCMV-SB100X, pCMV-mSB, pCMV-Flpe and pUC19 were used. Provided plasmid combinations were co-transfected and two days post-transfection cells were diluted and kept under selection pressure for two weeks. Error bars indicate standard deviation (n=3). *Significant difference between the group which received Flpe (+Flpe) and the group without Flpe (-Flpe) (p-value < 0.05).
(TIFF)

Figure S3 **Flpe recombinase does not enhance transposition efficiencies from AAV vector genomes.** (**a**) Transposon-substrates can be excised from AAV vector genome by Flpe mediated recombination. The transposon-donor vector AAV-transgene contains the transgene flanked by transposon derived inverted repeats (IR) and Flpe recombinase recognition sites FRT. This AAV vector was co-infected either with a Flpe encoding vector (AAV-mSB-Flpe) or as a control with the vector AAV-eGFP. After co-transduction into Huh7-cells (left panel) and HEK293-cells (right panel), a 700 bp fragment is PCR amplified if circularization occurred (black arrows). Red arrows depict PCR primer binding sites for the circularization PCR. (**b**) SB100X-HEK293 and mSB-HEK293 cells were co-infected with the recombinant vector AAV-neo at MOI 10,000 and the previously published Flpe encoding high-capacity adenoviral vector Ad5-mSB-Flpe. Two days post-infection cells were diluted and kept under selection pressure for two weeks. Error bars indicate standard deviation (n=3). “n.s.”: not significant, no significant difference compared to the control group (p-value > 0.05).
(TIFF)

Table S1 **Oligonucleotides used in this study.**
(DOC)

Table S2 **Summary of the molecular analysis of cells which received the inactive transposase (mSB).**
(DOC)
