# Correction: Hybrid Adeno-Associated Viral Vectors Utilizing Transposase-Mediated Somatic Integration for Stable Transgene Expression in Human Cells

**DOI:** 10.1371/annotation/058e5bbd-d902-4936-b763-a5a3c5cc995e

**Published:** 2013-11-19

**Authors:** Wenli Zhang, Manish Solanki, Nadine Müther, Melanie Ebel, Jichang Wang, Chuanbo Sun, Zsuzsanna Izsvak, Anja Ehrhardt

The funding source "E-rare project Transposmart" was missing from the Funding section. Please see the corrected Funding statement here:

DFG grant SPP 1230 (A.E.), the E-rare project Transposmart (A.E. and Z.I.), and EU Framework Programme 7 (Persistent Transgenesis)(A.E.). W.Z. was supported by a fellowship from the Chinese Scholarship Council (CSC) in cooperation with Northwestern A&F University, Yangling, China. The funders had no role in study design, data collection and analysis, decision to publish, or preparation of the manuscript. 

