# Supplementary figures and images for: Hybrid Adeno-Associated Viral Vectors Utilizing Transposase-Mediated Somatic Integration for Stable Transgene Expression in Human Cells
Source: PLoS One. 2013 Oct 8;8(10):e76771. doi: 10.1371/journal.pone.0076771 (PMC3792901; doi:10.1371/journal.pone.0076771)

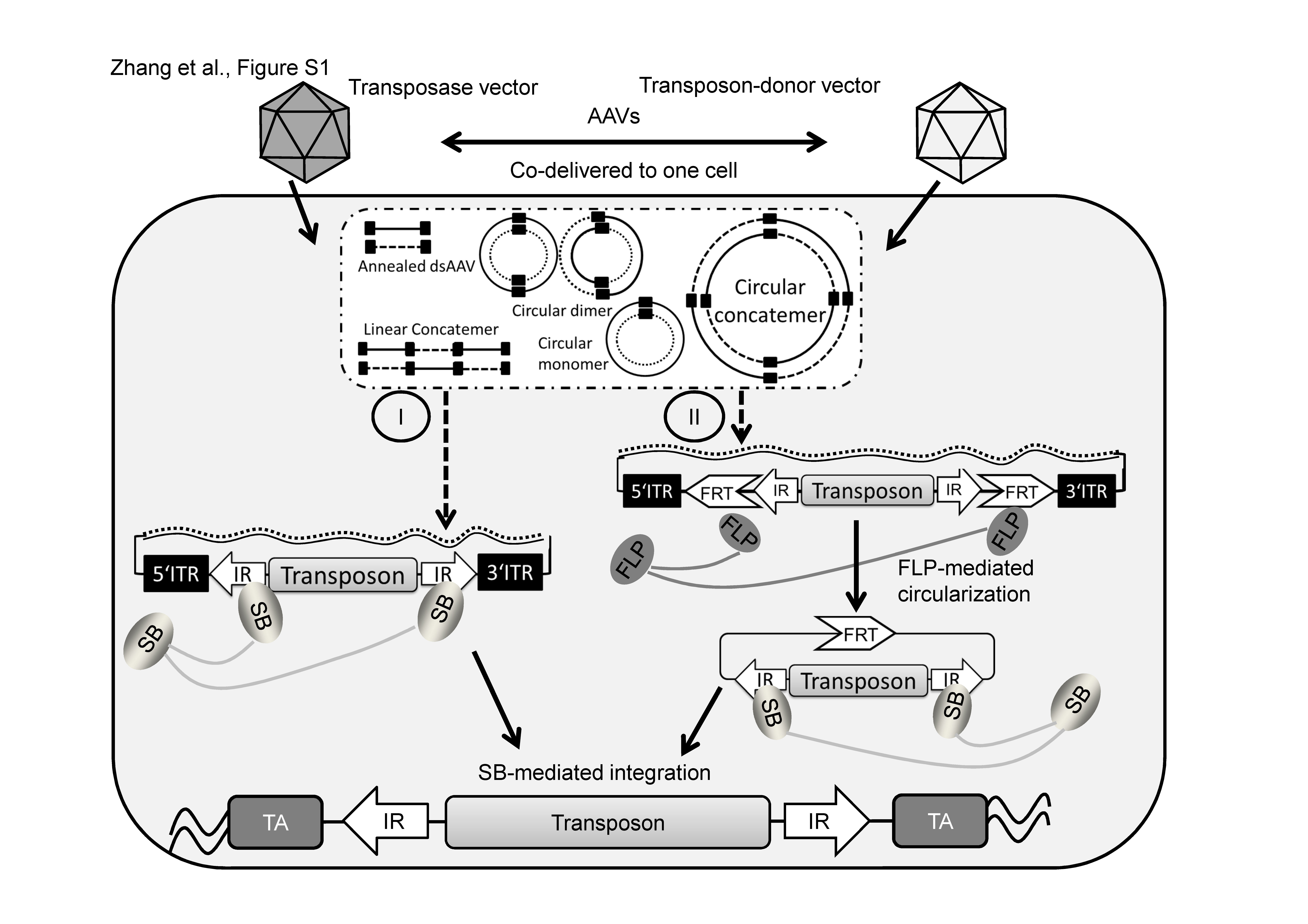

Supplement: Figure S1 — Molecular design of the AAV/SB hybrid vector system. For somatic integration cells are simultaneously infected with the transposase-vector and the transposon donor vector. After entering the cell, AAV vector genomes form different molecular forms including circular monomers, dimers, and concatemers as well as linear monomers and concatemers. Two strategies were pursued to mobilize the transposon form the AAV vector representing the transposon-donor: (I) the transposon flanked by inverted repeats (IR, white horizontal arrows) is directly mobilized from the AAV vector genome by the SB transposase protein provided in trans, and integrated into a genomic target site (TA dinucleotide) (II). After entering the cell, Flpe recombination excises and circularizes the transposon from the various forms of the AAV vector genomes by recognizing the FRT sites contained in the transposon donor vector. Subsequently, the transposon flanked by IRs is mobilized from the circular intermediate by the SB transposase protein provided in trans. As a last step the transposon integrates in the host genome (waved black lines) into the genomic target site (TA-dinucleotide). (TIFF) [file pone.0076771.s001.tiff]

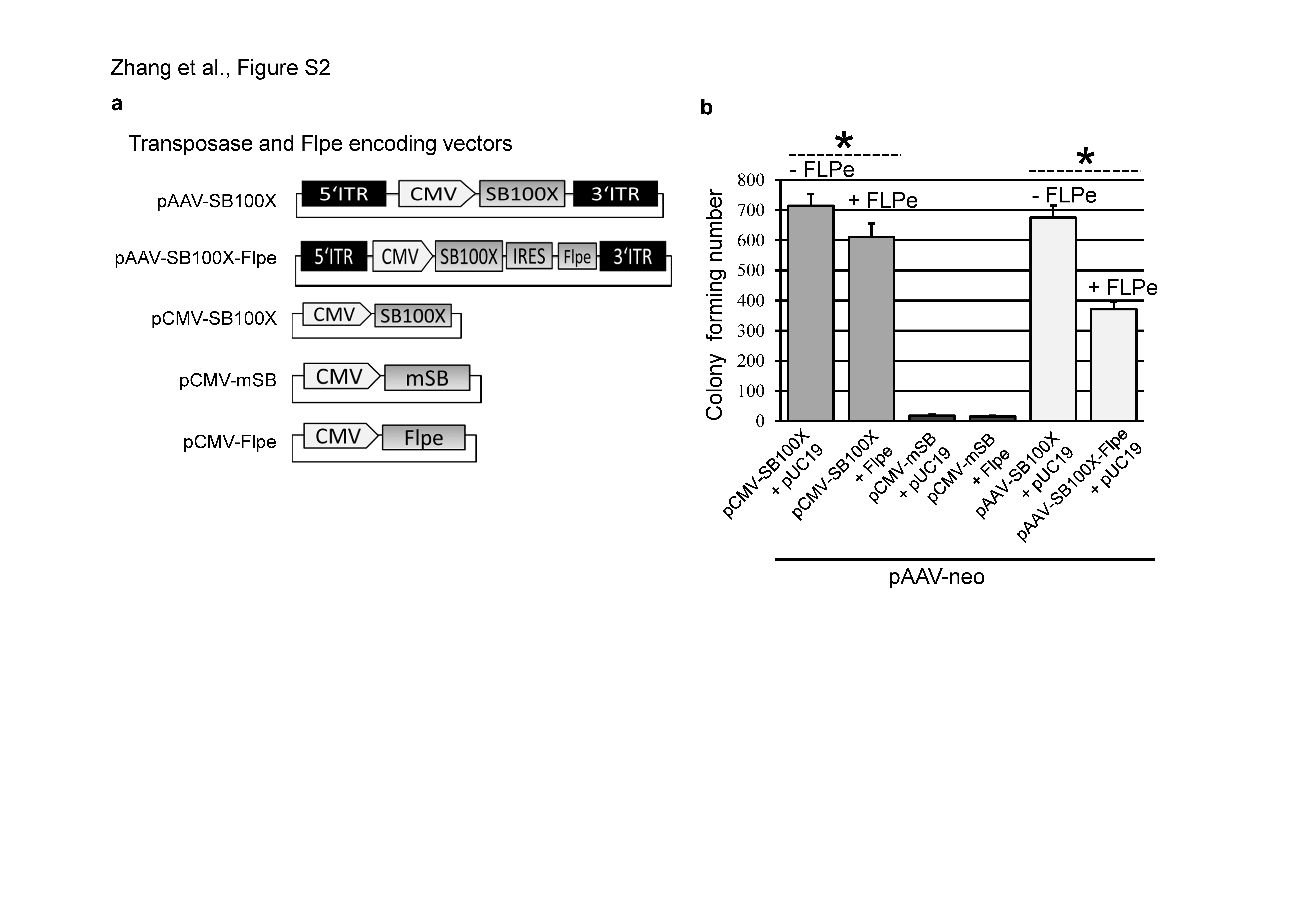

Supplement: Figure S2 — The effect of Flpe expression on transposition efficiencies. Colony forming assays were performed in HeLa-cells. (a) The Sleeping Beauty transposase encoding AAV plasmids pAAV-SB100X, pAAV-SB100X-Flpe, express transposase under the control of the cytomegalovirus promoter (CMV). Plasmids pCMV-SB100X and pCMV-mSB also encode active and inactive SB transposase genes and the plasmid pCMV-Flpe expresses codon optimized Flp recombinase under the control of the CMV promoter. (b) Colony forming assays were performed in HeLa-cells. Equal molar ratios for plasmids pAAV-SB100X, pAAV-SB100X-Flpe, pCMV-SB100X, pCMV-mSB, pCMV-Flpe and pUC19 were used. Provided plasmid combinations were co-transfected and two days post-transfection cells were diluted and kept under selection pressure for two weeks. Error bars indicate standard deviation (n=3). *Significant difference between the group which received Flpe (+Flpe) and the group without Flpe (-Flpe) (p-value < 0.05). (TIFF) [file pone.0076771.s002.tiff]

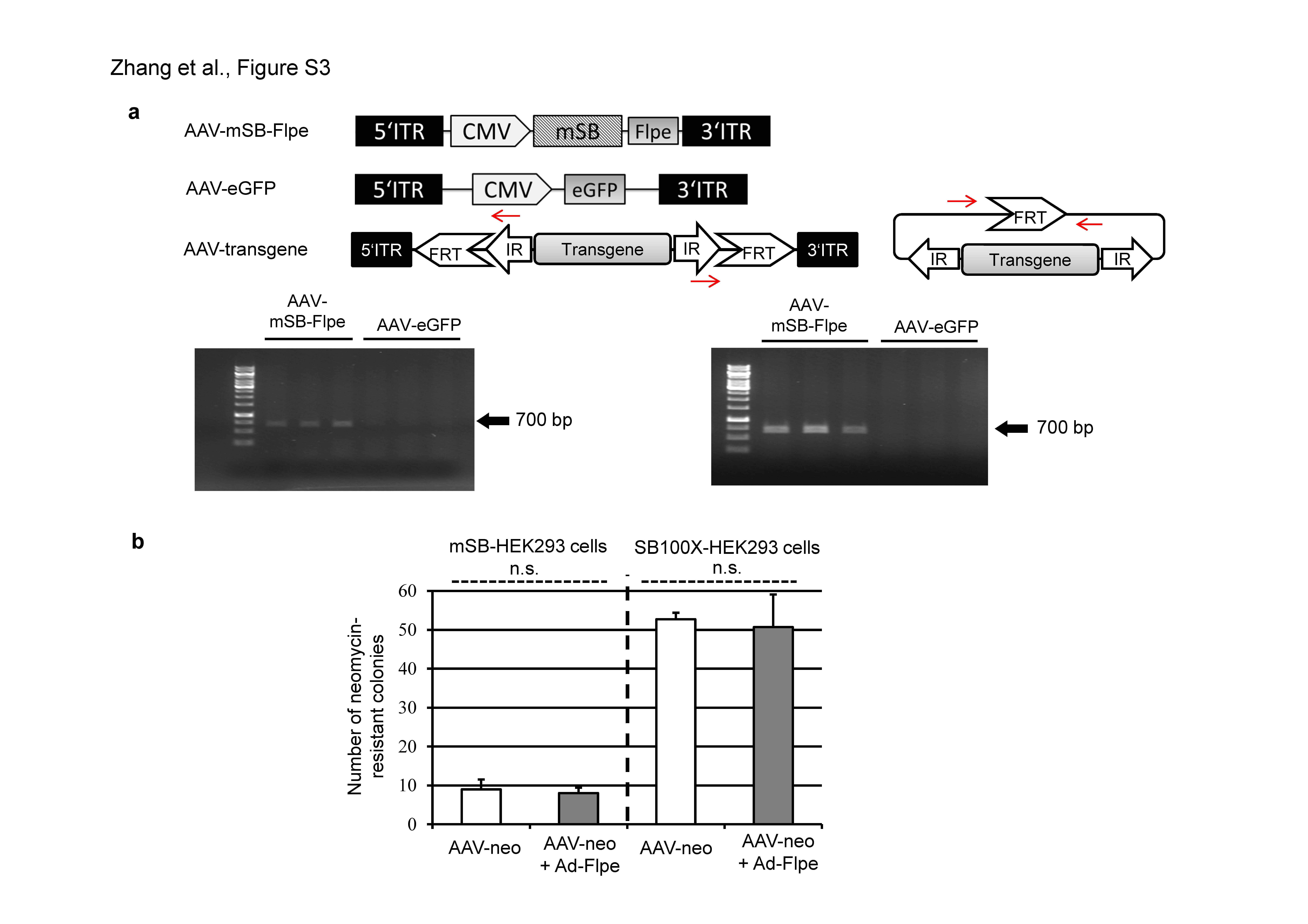

Supplement: Figure S3 — Flpe recombinase does not enhance transposition efficiencies from AAV vector genomes. (a) Transposon-substrates can be excised from AAV vector genome by Flpe mediated recombination. The transposon-donor vector AAV-transgene contains the transgene flanked by transposon derived inverted repeats (IR) and Flpe recombinase recognition sites FRT. This AAV vector was co-infected either with a Flpe encoding vector (AAV-mSB-Flpe) or as a control with the vector AAV-eGFP. After co-transduction into Huh7-cells (left panel) and HEK293-cells (right panel), a 700 bp fragment is PCR amplified if circularization occurred (black arrows). Red arrows depict PCR primer binding sites for the circularization PCR. (b) SB100X-HEK293 and mSB-HEK293 cells were co-infected with the recombinant vector AAV-neo at MOI 10,000 and the previously published Flpe encoding high-capacity adenoviral vector Ad5-mSB-Flpe. Two days post-infection cells were diluted and kept under selection pressure for two weeks. Error bars indicate standard deviation (n=3). “n.s.”: not significant, no significant difference compared to the control group (p-value > 0.05). (TIFF) [file pone.0076771.s003.tiff]
