# Supplementary material for: Hybrid Adeno-Associated Viral Vectors Utilizing Transposase-Mediated Somatic Integration for Stable Transgene Expression in Human Cells
Source: PLoS One. 2013 Oct 8;8(10):e76771. doi: 10.1371/journal.pone.0076771 (PMC3792901; doi:10.1371/journal.pone.0076771)
Supplement: Table S1 — Oligonucleotides used in this study. (DOC) [file pone.0076771.s004.doc]

**Table S1. Oligonucleotides used in this study.**

| **Target/Name** | **Orientation** | **Sequence (5’ to 3’)** | **Reference/Notes** |
| --- | --- | --- | --- |
| AAV- ITR fwd | Forward | GGAACCCCTAGTGATGGAGTT |  |
| AAV- ITR rev | Reverse | CGGCCTCAGTGAGCGA |
| AAV-ITR probe |  | [6-FAM]CACTCCCTCTCTGCGCGCTCG [BBQ] | Real-time PCR |
|  |  |  |  |
| Flpe-fw-BamHI | Forward | ACCCAGGATCCACCATGCCACAAT TTG | Amplification of the Flpe gene |
| Flpe-rev-NotI | Reverse | TGTTTGTGGCGGCCGCTGCGTGTTTATGCTT |
|  |  |  |  |
| human B2M-F | Forward | TGCTGTCTCCATGTTTGATGTATCT | Real-time PCR |
| human B2M-R | Reverse | TCTCTGCTCCCCACCTCTAAGT |
|  |  |  |  |
| IRES-fw-XbaI/BclI | Forward | CAACATCTAGATGATCAATTCCGCCCCTCTC | Amplification of the IRES sequence |
| IRES-rev- XbaI/BamHI | Reverse | AAGAATCTAGAGGATCCCGGTCATGGAAGG TC |
|  |  |  |  |
| neo-F | Forward | ACCAAGCGAAACATCGCATCGAG | Real-time PCR |
| neo-R | Reverse | GCGATACCGTAAAGCACGAGGAAG |
|  |  |  |  |
| SB-forw-XhoI | Forward | GATAACTCGAGATGGGAAAATCAAAAGAA | Amplification of the SB transposase gene |
| SB-rev-EcoRI | Reverse | CAGGTGAATTCCTAGTATTTGGTAGCATTG |
|  |  |  |  |
| SB-L | Forward | GGTGGCAGCATCATGTTGTG | Real-time PCR |
| SB-R | Reverse | CCTTCCTCATGATGCCATCTATT |
|  |  |  |  |
| SB100-for | Forward | GGAGGAAGAAGGGGGAGGCTT | To check SB transcription |
| SB100-rev | Reverse | GCCTCCCCCTTCTTCCTCCAA |

**References**

1. Aurnhammer C, Haase M, Muether N, Hausl M, Rauschhuber C, et al. (2012) Universal real-time PCR for the detection and quantification of adeno-associated virus serotype 2-derived inverted terminal repeat sequences. Hum Gene Ther Methods 23: 18-28.

2. Vandesompele J, De Preter K, Pattyn F, Poppe B, Van Roy N, et al. (2002) Accurate normalization of real-time quantitative RT-PCR data by geometric averaging of multiple internal control genes. Genome Biol 3: RESEARCH0034.
