# Supplementary material for: Hybrid Adeno-Associated Viral Vectors Utilizing Transposase-Mediated Somatic Integration for Stable Transgene Expression in Human Cells
Source: PLoS One. 2013 Oct 8;8(10):e76771. doi: 10.1371/journal.pone.0076771 (PMC3792901; doi:10.1371/journal.pone.0076771)
Supplement: Table S2 — Summary of the molecular analysis of cells which received the inactive transposase (mSB). (DOC) [file pone.0076771.s005.doc]

**Table S2. Summary of the molecular analysis of cells which received the inactive transposase (mSB).** Genomic DNAs of neomycin-resistant cells after selection were isolated and analysed by quantitative real-time PCR (qRT-PCR) using primers and probe detecting AAV-derived inverted terminal repeats (ITRs) and cDNAs of the two main functional genes neo (neomycin resistance gene) and SB (Sleeping Beauty transposase encoding gene). MOI: multiplicity of infection.

| Groups | Viral vector (MOI) | | Vector fate (Neo) | Genome copy numbers (per 1,000 cells) | | |
| --- | --- | --- | --- | --- | --- | --- |
|  | AAV-neo | AAV-mSB | Determined by plasmid rescue | neo | SB | 2xITR |
| 1 | 1,000 | 10,000 | transposition and vector insertion | 264.30±8.73 | 0.0695±0.0458 | 150.70±0.51 |
| 2 | 1,000 | 50,000 | vector insertion | 386.80±2.34 | 0.0531±0.0286 | 324.67±20.71 |
| 3 | 10,000 | 1,000 | vector insertion | 1181.57±105.56 | 0.0745±0.0196 | 128.91±3.66 |
| 4 | 10,000 | 10,000 | transposition | 656.51±26.42 | 0.1400±0,0187 | 113.62±9.01 |
